# Understanding a national increase in COVID-19 vaccination intention, the Netherlands, November 2020–March 2021

**DOI:** 10.2807/1560-7917.ES.2021.26.36.2100792

**Published:** 2021-09-09

**Authors:** Jet G. Sanders, Pita Spruijt, Mart van Dijk, Janneke Elberse, Mattijs S. Lambooij, Floor M. Kroese, Marijn de Bruin

**Affiliations:** 1 Corona Behavioural Unit, National Institute for Public Health and the Environment, Bilthoven, the Netherlands; 2 Department of Psychological and Behavioural Science, London School of Economics and Political Sciences, London, United Kingdom; 3 Department of Social, Health and Organizational Psychology, Utrecht University, Utrecht, The Netherlands; 4 IQ Healthcare, Radboud Institute for Health Sciences, Radboud University Medical Center, Nijmegen, the Netherlands

**Keywords:** intentions, beliefs, vaccine hesitancy, psychosocial determinants, COVID-19, The Netherlands

## Abstract

The intention to get the COVID-19 vaccine increased from 48% (November 2020) to 75% (March 2021) as national campaigning in the Netherlands commenced. Using a mixed method approach we identified six vaccination beliefs and two contextual factors informing this increase. Analysis of a national survey confirmed that shifting intentions were a function of shifting beliefs: people with stronger intention to vaccinate were most motivated by protecting others and reopening society; those reluctant were most concerned about side effects.

Mass vaccination against coronavirus disease (COVID-19) is an important tool to control the pandemic and recover from its consequences [1]. Vaccine hesitancy may hamper the effectiveness of vaccination programmes [2]. Knowledge of which factors are associated with vaccination hesitancy can guide efforts towards developing effective campaigns to increase acceptance of the COVID-19 vaccine and maximise uptake [3-5]. A nationally representative survey in the Netherlands showed that vaccination intention increased substantially from 48% in November 2020 to 75% in January 2021 (Figure 1) when the national COVID-19 vaccination campaign commenced [6,7]. Our aim was to determine which psychosocial factors were associated with this shift in intentions to get the COVID-19 vaccination.

**Figure 1:**
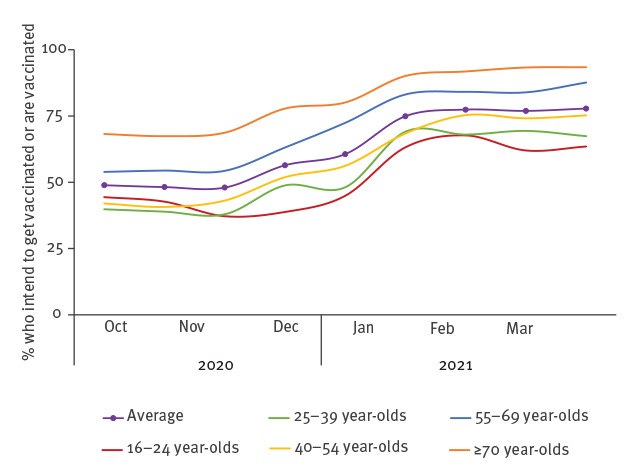
Intention to get vaccinated against COVID-19 according to a national trend survey, November 2020 to January 2021, the Netherlands^a,b^

COVID-19: coronavirus disease.

^a^ The national trend survey represents a cross-sectional representative sample of the Dutch population. Samples ranged from 4,890 and 5,262 per Wave (average 5,060) with a response rate of 52%. All participants who completed the survey, responded to the question. Confidence intervals fall within 1.5% of the average. Data was retrieved and adapted from: https://coronadashboard.government.nl/landelijk/vaccinaties.

^b^ Participants were asked about their vaccination status and intentions if they had not yet been vaccinated. Participants were invited to vaccinate over this time-period using a staggered roll-out according to prioritisation criteria such as age, health status and essential worker status. Vaccination rates increased over time. To represent pro-vaccination intention accurately for the Dutch population, we use a combined outcome variable, which incorporates (i) those with a positive vaccination intention and; (ii) those who have already been vaccinated (reflecting a pro-vaccination intention).

This figure is adapted from: https://coronadashboard.government.nl/landelijk/vaccinaties.

## Beliefs and intentions

We identified reasons for different vaccination intentions through open-ended responses to a question about reasons behind one’s vaccination intention in a national cohort survey and qualitative interviews.

As part of a six-weekly national cohort survey we asked 64,170 participants to indicate their vaccination intention during Wave 8 of the survey in November 2020 [8]. All 64,170 participants (see Supplementary materials 1 for demographic details) provided a reply through the closed-ended question on their vaccination intention (the exact wording of this question was: “*If there is a vaccine against the coronavirus, will you get vaccinated?*”); if participants indicated that they intended to vaccinate (n = 32,471) or did not intend to vaccinate (n = 7,530) they were shown an adjacent and optional open ended question asking them to write about their reasons for their vaccination intention. Of these 11.7% (n = 7,106) provided a response to the open-ended question. We analysed all the open-ended responses provided by the 2,292 participants who indicated to have the intention to get vaccinated and all open-ended responses by the 2,393 participants with no intention to get vaccinated (see Supplementary materials 2 for details).

Next, we conducted (in January 2021) 60 semi-structured telephone interviews over a 4-day period among participants who indicated to be uncertain or to have no intention to get vaccinated in Wave 9 of the cohort study in December 2020 (see Supplementary materials 3 for methodological and participant details). The aim of the interviews was to capture concerns and beliefs for this population in a time where we observed intentional switching. Interviews were transcribed verbatim (under supervision of PS) and thematic analysis [9] was performed (by PS and JE; see Supplementary materials 4 for results). Insights were pooled across the two datasets.

We identified six common beliefs about COVID-19 vaccination: (i) concerns about short-term side effects; (ii) concerns about long-term side effects; (iii) personal vaccination will protect others (this includes a sense of moral duty); (iv) personal vaccination will protect oneself; (v) trust in science or institutions; (vi) vaccination is key to reopening society (Table 1).

**Table 1:** Dominant beliefs and contextual factors in relation to COVID-19 vaccine intention identified in open-ended responses and interviews distilled into statements used in subsequent waves of the national cohort survey, the Netherlands, November and December 2020

Beliefs and contextual factors	Open-ended answers (Wave 8; Nov 2020)		Interviews (Wave 9; Dec 2020)		Statements, questions and response options used in cohort survey^a^ (Wave 10; Feb 2021 and Wave 11; Mar 2021)
	Yes, because…	No, because…	Drivers	Barriers	
Belief 1: Concerns about short-term side effects	Na	Potential side effects: “*First I want to know if there are really no side-effects.*” Participants indicated that they did not wish to get vaccinated because of their physical condition “*I have allergies.*”, “*I always get sick from vaccinations.*”, “*I can’t in my current condition.*”	Na	Current side effects, for example in relation to medication use. Recommendation from health professional were also mentioned as deciding factor: “*I let myself be guided by the recommendation of my cardiologist. I am not against vaccinations, but I am also a heart patient and any potential side- effects are, therefore of critical importance.*”	I am scared of potential side effects of the vaccine. Response options: Completely disagree; disagree; neutral; agree; completely agree
Belief 2: Concerns about unknown long-term side effects	Na	As a result from rapid development participants reported that long term consequences are a concern, in particular in relation to fertility “*We don’t know about pregnancy yet.*”; Participants mentioned that they preferred to wait a bit longer until more is known.	Na	Long-term consequences: “*If it is only effective for three months, then I won’t get it*”. Examples: medications which were discovered to have negative side-effects later. Some women expressed concerns about potential effect on fertility. Others employed the wait and see approach: one is inclined not to vaccinate until more knowledge on long-term side effects gives greater confidence in the vaccine.	I am scared of possible unknown long-term effects of the vaccine. Response options: Completely disagree; disagree; neutral; agree; completely agree
Belief 3: Personal vaccination will protect others (moral duty)	Protecting (vulnerable) loved ones was an important reason “*It feels safer for my husband*.” “*My parents are old.*”; “*To protect my family.*” Some people noted protection of others as a moral duty “*It’s just important*”; “*I am doing this for the social good.*”	Na	To protect others, including for the greater good. “*I want the vaccine to contribute to protecting others.*”	Na	If I am vaccinated, I protect others from the coronavirus. Response options: Completely disagree; disagree; neutral; agree; completely agree
Belief 4: Personal vaccination will protect oneself	Protect oneself for fear of getting sick or risk trade-off between COVID-19 and vaccination “*Better safe than sorry.*”	No need to protect oneself. In risk trade off, no or little fear of illness “*Coronavirus is not dangerous for me.*”; “*Chances that I get ill are small.*”; “*I prefer getting corona than getting a jab.*”	“*By getting vaccinated I protect myself and regain some freedom.*”	Na	If I get vaccinated, I am protected from the coronavirus. Response options: Completely disagree; disagree; neutral; agree; completely agree
Belief 5: Trust in science or institutions	Trust in national institutions “*If it’s approved it should be fine.*” No different from other vaccines.	Generalised lack of trust “*I don’t trust it.*”; “*No government will invade my body.*”; Speed of development could not have allowed for appropriate testing: “*I think it went too fast.*”; “*It was brought to the market too quickly.*”	Trust in other organisations. Comparison with other vaccines: “*I wouldn’t usually question this.*”; confidence that government acts for the good of society and that the scientific community acts responsibly and carefully.	Concerns over rapid development raises questions about safety of the vaccine and potential (long-term) side effects; distrust of the mRNA vaccines, as this is a new type of vaccine.	If the vaccine has been approved on the Dutch market, I believe that it is safe. Response options: Completely disagree; disagree; neutral; agree; completely agree
Belief 6: Vaccination is key to reopening society	Wanting to get vaccinated to fight the pandemic “*It will help to slow the virus and stop the pandemic.*”; or to reopen society: “*So we can go back to normal.*”	Na	Contributing to a way out of the crisis to be able to have more social contacts, hug or take part in non-essential activities again.	Na	If I get vaccinated, I contribute to a way out of the corona crisis for the Netherlands. Response options: Completely disagree; disagree; neutral; agree; completely agree
Contextual factor 1: Social context	Na	Na	An increasing/high vaccination rate works as a driver (greater confidence in safety/effectiveness) “*The more people that are vaccinated, the safer it is for me to also be vaccinated.*”	Increasing/high vaccination rate works as a barrier. If already high: “*I no longer need to do it.*”. This is also known as “*free-riding*”.	Most of my friends and family have been vaccinated against corona or are planning to do so. Response options: Completely disagree; disagree; neutral; agree; completely agree
Contextual factor 2: Cue to action	Invitation letter: “*I am being asked to.*”; or through trusted messengers: “*My doctor says I should.*”	Na	Invitation letter: “*I will make my decision when it becomes relevant.*”; information from trusted individuals (medical experts), e.g. on TV. “*If I see a reputable doctor on TV who answers a number of questions, then it makes me more relaxed and, despite my doubts, also gives me confidence to be vaccinated.*”	Na	Have you already received an invitation, or are you due to be vaccinated against the coronavirus?^b^ Response options: Yes; no; I don’t know^c^.

COVID-19: coronavirus disease; Na: not applicable; TV: television.

^a^ Wording of the statements and questions used in the national survey was adjusted in language to accommodate colloquial referral to COVID-19.

^b^ We noted both the invitation letter and trusted public messengers as a salient environmental cues, however opted to incorporate only an item on invitation letters into the cohort survey. This was for two reasons: we had restrictions in the number of items that could be incorporated, and campaigning and influence of trusted messengers we believed may be harder to retrace or identify for the individual. We therefore opted to incorporate only the cue to action of the invitation letter as a single item statement in the cohort survey.

^c^ Only 26 participants answered *“I don’t know”*. Due to the small sample size this group was removed from the analysis and the variable was entered as a dichotomous variable ‘yes/no’ in the regression model.

While the choice to get a vaccine is dichotomous, we found that vaccination intention functioned on a continuum, coupled with distinctive belief profiles: at the reluctant end barriers dominated (Beliefs 1 and 2). As the share of favourable arguments (Beliefs 3−6) increased respondents’ intentions moved from ‘probably not’, to ‘probably’, and finally to ‘definitely’ getting vaccinated.

Next to these six beliefs, we observed two contextual factors that played an important role in changing intentions i.e. the social context and cue to action.

With regards to social context, participants frequently mentioned not wanting to be the first to get vaccinated (*“I am not a guinea pig“*). For some participants rising vaccination rate was seen as a driver to getting vaccinated (a signal for safety and effectiveness), whilst for others this was seen as a barrier (if high *“I no longer need to do it“*). The latter can be linked to the ‘free-riding’ phenomenon [10]).

For cue to action, some participants postponed their decision until they would receive their official invitation (in the form of a letter). Beliefs were updated periodically, informed by (non-persuasive) pro-choice information on side effects by public individuals. National campaigns and targeted media reports by medical experts, or personal conversations with health professionals were named as decisive messengers.

Of the 60 interviewees, 18 (30%) had changed their vaccination intention from not intending to or uncertain of getting vaccinated, to intending to get vaccinated between survey completion and their interview (2-week gap). This was an indication that vaccination intention might not be fixed and might change over time.

## Beliefs predicting shifts in intention

**Figure 2:**
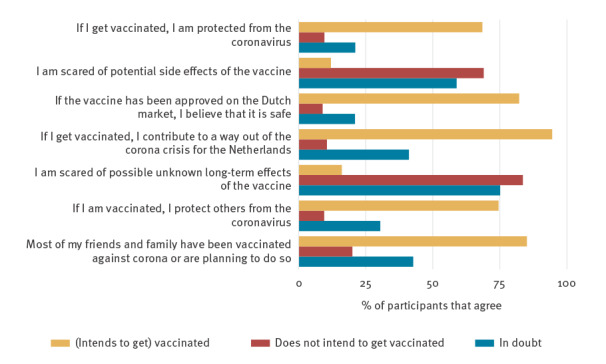
Percentage of participants who indicated to ‘agree’ or ‘completely agree’ for the vaccination belief statements separated by (who intends to get) vaccinated (yellow), those who do not (red) and those who are in doubt (blue), national cohort survey Wave 10, the Netherlands, February 2021 (n = 52,400)

The six beliefs related to COVID-19 vaccination and two contextual factors were incorporated into Wave 10 of the cohort survey (February 2021). Vaccination beliefs and contextual factors were assessed using single item measures (see Table 1 for exact statements and response options). Descriptive statistics for the 52,400 respondents confirmed that vaccination beliefs display on a continuum (Figure 2).

COVID-19: coronavirus disease.

Colloquial expressions were used in the belief statements to ease public understandings.

To identify which beliefs were most relevant in the context of COVID-19 vaccination intention switching, we conducted a multinomial logistic regression of the vaccination beliefs (continuous variables) and contextual factors (social context as continuous, cue to action as dichotomous) in Wave 10 (February 2021) for participants who had been uncertain at Wave 10 and switched to ‘yes’ or ‘no’ relative to those who did not (‘still uncertain’) in Wave 11 (March 2021; n = 3,383; Table 2). Response options for vaccination beliefs and the social context statements were ordinal on a 1−5 Likert scale. These were incorporated into the regression model as continuous variables.

**Table 2:** Multinomial logistic regression of vaccination beliefs and contextual factors for participants ‘uncertain’ at Wave 10 and who switched to ‘yes’ or ‘no’ relative to those who did not in Wave 11, national cohort survey, the Netherlands, February–March 2021 (n = 3,383)

Beliefs (Wave 10)	Switched intention to get vaccinated (n = 1,197)				Switched intention to not get vaccinated (n = 258)		
	Adjusted OR	95% CI		p value	Adjusted OR	95% CI	p value
I am scared of potential side effects of the vaccine.	0.89	0.80–0.98		0.02	1.02	0.86–1.21	0.83
I am scared of possible unknown long-term effects of the vaccine.	0.90	0.81–1.00		0.05	1.06	0.87–1.29	0.54
If I get vaccinated, I am protected from the coronavirus.	1.10	0.99–1.22		0.09	1.18	0.99–1.40	0.07
If I am vaccinated, I protect others from the coronavirus.	1.09	0.98–1.22		0.10	0.89	0.75–1.07	0.22
If the vaccine has been approved on the Dutch market, I believe that it is safe.	1.31	1.16–1.47		<.001	0.71	0.59–0.86	<.001
If I get vaccinated, I contribute to a way out of the corona crisis for the Netherlands.	1.56	1.39–1.76		<.001	0.80	0.67–0.96	0.02
**Contextual factors**							
Most of my friends and family have been vaccinated against corona or are planning to do so (social context, Wave 10).	1.15		1.03–1.28	0.01	0.96	0.80–1.14	0.60
Have you received an invitation, or is your vaccination against the coronavirus due? Cue to action, Wave 11.	4.25		3.29–5.50	<.001	1.64	1.04–2.59	0.03
**Sex**							
Female	Reference						
Male	1.04		0.87–1.25	0.68	0.88	0.63–1.21	0.42
**Age**							
≥70 years old	Reference						
55–69 years old	0.71		0.54–0.94	<.001	0.71	0.41–1.22	0.22
40–54 years old	0.49		0.37–0.65	<.001	0.89	0.52–1.52	0.68
25–39 years old	0.40		0.29–0.55	<.001	1.27	0.73–2.21	0.40
16–24 years old	0.40		0.23–0.70	0.02	0.91	0.35–2.32	0.84
**Education**							
Higher education	Reference						
Secondary education	1.04		0.87–1.24	0.65	1.34	1.00–1.79	0.05
Primary education or lower vocational education or no education	0.91		0.70–1.17	0.46	1.38	0.91-2.10	0.13

CI: confidence interval; OR: odds ratio.

Reference: those who remain in doubt (n = 1,928)

Those who switched from uncertain in their intention to get vaccinated (n = 1,197) had less uncertainty about short-term (adjusted Odds Ratio (aOR) = 0.89) or long-term side effects (aOR = 0.90), reported higher institutional trust (aOR = 1.31) and stronger belief that vaccination protects others (aOR = 1.56) in the wave prior to their switch, relative to those who were still in doubt (n = 1,928). They also reported having more people in their social environment who had been vaccinated (aOR = 1.15). Those who switched from being uncertain to having no intention to get vaccinated (n = 258) oppositely report lesser institutional trust (aOR = 0.71) and weaker belief that vaccination protects others (aOR = 0.80) in the wave prior to their switch, than those who remained in doubt. As expected from the qualitative results, in both groups the switch in vaccination intention related strongly to receiving the invitation to get vaccinated (aOR = 4.25 and aOR = 1.64).

## Ethical statement

The study does not meet the requirement as laid down in the Law for Research Involving Human Subjects (WMO) and was therefore exempted from formal ethical review. Informed consent was provided by all participants.

## Discussion

Based on these findings, and its co-occurrence with the national increase in pro-vaccination intention in the Netherlands, we put forward three pillars for national pro-vaccination informational campaigns.

Firstly, as people’s beliefs inform their intentions, informational campaigns should provide reliable (not persuasive) information for informed autonomous choice. This may contain individually tailored advantages (e.g., protective benefits toward self/others, or a staged release of the COVID-19 measures). The campaigns may also benefit from presenting disadvantages of vaccination (e.g., risks of side effects) in trade-off with the risks of not vaccinating against COVID-19. Finally, campaigns may benefit from information on how a vaccine was developed so quickly and that no compromises were made on quality and procedure should also be communicated and which steps are being taken to monitoring it’s safety to share these transparently [11].

Secondly, people may periodically update their beliefs and intentions and it is important to provide support throughout the choice process. This may concern information about vaccination through reliable channels i.e., medical experts on mass or social media, or targeted very brief advice (VBA) from general practitioners or professional patient associations [12]. Such a conversation with a reliable advisor or doctor may be even more effective if local trust in government (and its institutions) is low [13]. Accurate and up-to-date information on numbers of people who have been vaccinated and adverse events should be provided throughout the vaccination campaign. Getting vaccination should be linked to other beliefs, such as the duty to protect others or getting out of crisis.

Thirdly, people may have the intention, but not follow through (also called the intention–behaviour gap [21,22];) so in addition to a focus on people’s beliefs vaccine uptake needs to be easy and accessible. This may be realised through use of localised or mobile vaccination sites [14,15] with walk-in or drive-through appointments [16,17], sending reminder messages or letters to get vaccinated (cue to action; consider timing, intensity and content of messages [20]), or by facilitating peer-to-peer social media sharing to set the social norm [18,19].

## Conclusions

In March 2021, approximately one in five people in the Netherlands reported having no intention or being unsure of whether to get vaccinated against COVID-19. As vaccine uptake is likely to be lower than vaccination intention, this is still worryingly high considering the impact of COVID-19 on day-to-day life. Our findings demonstrate that (i) vaccination intention functions on a spectrum and can change over time; (ii) the actual choice to get vaccinated or not happens at the point of official invitation; (iii) the directional shifts in intention can be predicted by patterns in beliefs and the social norm prior to the choice process. We expect that these patterns hold beyond the Dutch context, and possibly beyond this pandemic. Our results indicate that vaccination campaigns could be a decision aid to those at a decision point and increase chances of reaching critical vaccination levels.

## Supplementary Data

824000 Supplement
