# Supplementary material for: Understanding a national increase in COVID-19 vaccination intention, the Netherlands, November 2020–March 2021
Source: Euro Surveill. 2021 Sep 9;26(36):2100792. doi: 10.2807/1560-7917.ES.2021.26.36.2100792 (PMC8431991; doi:10.2807/1560-7917.ES.2021.26.36.2100792)
Supplement: Supplement [file 21-00792_SANDERS_Supplement.pdf]

Supplementary materials

This supplementary material is hosted by Eurosurveillance as supporting information alongside the article 'Understanding a national increase in COVID-19 vaccination intention, the Netherlands, November 2020–March 2021' on behalf of the authors who remain responsible for the accuracy and appropriateness of the content. The same standards for ethics, copyright, attributions and permissions as for the article apply. Supplements are not edited by Eurosurveillance and the journal is not responsible for the maintenance of any links or email addresses provided therein.

S1. Table of demographic characteristics for participants in Wave 8

	Wave 8 (n=64,170)
Gender	
Male	21,854 (34.1%)
Female	42,233 (65.8%)
Other	83 (0.1%)
Age	
70+	10,545 (16.4%)
55-69	19,651 (30.6%)
40-54	16,636 (25.9%)
25-39	14,062 (21.9%)
16-24	3,276 (5.1%)
Education	
Higher education	37,687 (58.7%)
General upper secondary education	18,453 (28.8%)
Primary education and general lower secondary education	7,244 (11.3%)
Unknown/missing	786 (1.2%)

S2. Analysis of reasons for survey participants' vaccination intentions, Wave 8

Purpose

We set out to determine which beliefs were held by those who did or did not intend to get vaccinated against the coronavirus.

Method

In the 8th wave of the national cohort survey (11-15 November 2020), participants could select a number of options in response to the question: 'Would he or she choose to be vaccinated if a vaccine against coronavirus was to be available soon.' The available options were: 'yes, because...' (n=32,471), 'yes, but I would first like to know whether I have already had coronavirus' (n=2998), 'no, because I have already had coronavirus' (n=427), 'I do not know yet', or 'no, because...' (n=7530). Respondents who selected the first or the last option could write down reasons for their selection in a blank text field. Of these 11.7% (n = 7,106) provided a response to the open-ended question. We analysed all the open-ended responses provided by the 2,292 participants who indicated to have the intention to get vaccinated and all open-ended responses by the 2,393 participants with no intention to get vaccinated. The main results are summarised below.

Results

Wanting to get vaccinated to protect oneself and others

The most frequently mentioned reason for getting vaccinated was to protect oneself, one's family and close friends, and/or (vulnerable) others. The vaccine is described to provide a feeling of 'safety'. In this context, specific mention was sometimes made of the fact that the participant was at risk, or had a profession which involved seeing many or vulnerable individuals.

Some indicated that they had weighed-up the risk of getting vaccinated with the risk of becoming infected with COVID-19. Vaccination seemed the better option to them (”better safe than sorry", "otherwise you could get very sick"). Some indicated that they wanted to get vaccinated for fear of becoming sick ("I'm afraid outside", "I'm scared of getting sick"). Few indicated that they would not (yet) get vaccinated for altruistic reasons (”others need it more”).

Wanting to get vaccinated to fight the pandemic

People often reported wanting to get vaccinated in order to fight the pandemic ("slowing the virus, fighting the pandemic"), or people saw vaccination as the right thing to do ("important", "moral obligation"). Vaccination was also mentioned as a way of being able to roll back COVID-19 safety measures.

Not wanting to get vaccinated because of possible side effects (resulting from rapid development)

Amongst the most frequent reasons not get vaccinated were that the vaccines were developed quickly and would not have been tested properly ("I think it went too fast", "brought to market much too quickly"). There were concerns about possible side- and other effects in the short and long term ("I first want to know if there really are no side-effects", "we don't yet know what

effect it has on pregnancy”) as well as the safety and reliability of the vaccines (“I don’t trust it”). People said that they did not “want to be a guinea pig” or that they preferred to wait a bit longer until more is known. In addition, there was a group of people who indicated that they did not want to get vaccinated as a matter of conviction (“I’m against vaccination”, “no vaccine in my body”).

Not wanting to get vaccinated because of minimal risk of becoming sick

Amongst those indicating they did not want to get vaccinated, some participants also reporting trading off the cost and benefit of vaccinating and getting sick with COVID-19 – in direct opposition to the previous sentiment – they decided that the latter was less dangerous (“coronavirus is not dangerous”, “I’m not afraid of coronavirus”, “I prefer getting corona to getting jabbed”), or that the risk of becoming sick was small (“the change I will get sick is small”).

One’s own poor health can be a reason for wanting to get vaccinated or not

Underlying conditions were also mentioned as a reason for not wanting to get vaccinated (“65+ years”, “I’m vulnerable”, “I have other conditions”’). In the group of people who did not want to get vaccinated, some indicated that they did not wish to do so based on their physical condition (“allergies”, “I always get sick from vaccinations”). This was for some paired with advice provided by medical experts.

S3. Interview sampling strategy, participant demographics topic guide Wave 9

We aimed to conduct 60-70 interviews in a 4-day period. All respondents to the cohort study Wave 9 were asked whether they were willing to participate in further research, such as partaking in interviews or focus groups, on topics related to Covid-19 and behavioural science. Participants were only contacted to partake in interviews if they had indicated that they were willing to partake in further research. Respondents who had participated in a previous round of interview were not eligible to partake. This generated a pool of possible invitees. To address the research, we used the following selection criteria for interview invitation. First, we identified respondents who had indicated ‘I do not know yet’, ‘no, because I have already had coronavirus’, or ‘no, because...’ in response to the question ‘Would you choose to be vaccinated if a vaccine against coronavirus is available soon?’ in the cohort survey. We opted to interview participants who has indicated ‘no, because…’ in addition to the participant pool ‘I don’t know’ to review the connection and possible overlap between held beliefs and vaccination intentions. Second, we used two selection criteria to create ten groups of potential respondents representing across the two demographic variables considered to be of high importance to vaccination beliefs: across the five age strata and two level of education:

Strata of participants eligible for interview by two demographic variables used for interview.

	Higher education	general and primary education
16 - 24	24	34
25 - 39	414	218
40 - 54	592	353
55 - 69	399	362
70+	120	131

Based on an estimated 50% uptake, 15 eligible individuals were invited to partake at random per group. These participants were invited to partake via email and phoned to schedule for interview based on their availability during the 4-day window. In total 60 interviews were conducted. See the demographic information of participants below:

Demographic characteristics of participants that were interviewed

	Round 9 (n=60)
Gender	
Male	22 (36.7%)
Female	38 (63.3%)
Other	0
Age	
70+	12 (20.0%)
55-69	12 (20.0%)
40-54	11 (18.3%)
25-39	12 (20.0%)

16-24	13 (21.7%)
Education	
Higher education	28 (46.6%)
General upper secondary education	23 (38.3%)
Primary education and general lower secondary education	8 (13.3%)
Unknown/missing	1 (1.7%)

The 8 interviewers were given detailed instructions, and used the below Topic Guide:

INTROTEKST: Goedendag, u spreekt met [.….] van het RIVM. Ik bel in het kader van het onderzoek naar de coronamaatregelen dat het RIVM samen met de GGD uitvoert. U heeft pas geleden een vragenlijst hierover ingevuld en daarin aangegeven dat u bereid bent om mee te doen aan een verdiepend onderzoek. Dat waarderen wij zeer! We hebben u een paar dagen geleden hier ook over gemaild. Het verdiepende onderzoek houdt in dat we mensen telefonisch willen interviewen over vaccineren. Een gesprek duurt maximaal 30 minuten. Zou u mee willen doen?
Zo ja, schikt het nu om even te bellen? [stilte] Of wilt u liever dat ik op een later moment terug bel?
Zo nee, helaas, maar geen probleem. Ik wens u verder nog een fijne dag [bel volgende respondent van je lijstje].
Fijn dat het schikt/fijn dat we nu even kunnen bellen.
Ik zou het interview graag willen opnemen. Vindt u het goed als ik het gesprek opneem? *[antwoord respondent afwachten]*. Dan begin ik de opname met nogmaals uw toestemming te vragen zodat die op de band staat.

[OPNAME STARTEN]
Ik heb de band gestart. Het is vandaag […datum…]. U heeft respondentnummer […] en u vindt het goed dat ik ons gesprek opneem; is dat correct? *[antwoord respondent afwachten]*

INTERVIEWVRAGEN

Algemeen (vragen voor ALLE respondenten)

INTRODUCTIETEKSTJE: *We gaan het vandaag hebben over de coronavaccins. Tijdens het interview zijn we vooral op zoek naar hoe u over het/de vaccin(s) denkt, over de rol die het volgens u speelt in de coronacrisis en welke zorgen of vragen er bij u leven. We doen dit om ervoor te zorgen dat de informatieverstrekking over het vaccin zo volledig mogelijk is, het is dus niet zo dat wij tijdens dit gesprek al eventuele vragen kunnen beantwoorden.*

1a. Hoe gaat het met u in de huidige situatie?
1b. Heeft u zelf sinds de uitbraak van het coronavirus corona gehad?
1c. Zijn er mensen in uw sociale omgeving die corona hebben of hebben gehad?

2. Hoe ervaart u de huidige lockdown?

3. In hoeverre volgt u de maatregelen die gelden tijdens deze lockdown? Het gaat dan om de maatregelen zoals bijv. de 1,5m afstand houden, zoveel mogelijk thuis werken, kinderen thuis houden, max. 2 bezoekers per dag ontvangen, niet reizen naar het buitenland.

Gevoelens en opvattingen over vaccinaties (vragen voor alle respondenten)

4. Wat komt er als eerste in u op als u aan het coronavaccin denkt?

5a. Welk gevoel roept het bij u op dat er nu een vaccin is tegen het coronavirus? *(vb antwoorden waar je aan kan denken: opgelucht, dankbaar, blij, argwaan, boos)*

5b. Kunt u dat toelichten?

6a. Hoe groot is volgens u de bijdrage van het vaccin in het bestrijden van de coronacrisis, op een schaal van 1 tot 10? 10 betekent van onschatbare waarde, een 1 is geen bijdrage.

6b. Kunt u dit toelichten?

7. In het verleden, tegen andere ziektes of vanwege reizen naar het buitenland, een vaccinatie laten toedienen? (*hier peilen of de respondent tegen vaccineren an sich is of specifieke bezwaren tegen het coronavaccin heeft*)

Vaccinatiebereidheid (vragen voor alle respondenten)

INTRODUCTIETEKSTJE: *Ik zag dat u in de vragenlijst heeft geantwoord dat u [zich niet wil laten vaccineren OF nog niet weet of u zich wilt laten vaccineren]*

8. Hoe denkt u daar op dit moment over?

Respondent wil zich WEL laten vaccineren

9. Wat zijn voor u de redenen om u te laten vaccineren tegen het coronavirus? *(vraag goed door!)*
(*Voor de interviewer: ga echt na welke opvattingen of misopvattingen er leven bij de respondent. Ga ook na of de opvattingen betrekking hebben op de werking van het vaccin, de bijwerkingen, de ontwikkeling ervan, het vaccinatiebeleid. Vraag door!)*

10a. Laten de mensen in uw omgeving, denk aan familie, vrienden, buren, collega's, zich vaccineren tegen corona

10b. Is dat wat uw omgeving kiest van invloed op uw keuze om u te laten vaccineren?

10c. Zo ja, welke mensen zijn voor u daarin belangrijk?

INTRODUCTIETEKSTJE: In de vragenlijst heeft u eerder aangegeven dat [u zich niet wilt laten vaccineren OF dat u nog niet weet of u zich wilt laten vaccineren]. Nu bent u daar wel toe bereid.

11. Wat heeft u van gedachten doen veranderen? *(voor de interviewer: we zijn hier op zoek naar de reden die de doorslag gaf om van 'twijfelaar' of 'nee-kiezer' naar voorstander te zijn gegaan)*

12a. Heeft u gebruik gemaakt van informatiebronnen bij uw keuze om u wel te laten vaccineren?

12b. Zo ja, welke?

Respondent wil zich <u>NIET</u> laten vaccineren

13. Wat zijn voor u de redenen om u niet te laten vaccineren tegen het coronavirus? (*Voor de interviewer: ga echt na welke opvattingen of misopvattingen er leven bij de respondent. Ga ook na of de opvattingen betrekking hebben op de werking van het vaccin, de bijwerkingen, de ontwikkeling ervan, het vaccinatiebeleid. Vraag door!)*

14a. Laten de mensen in uw omgeving, denk aan familie, vrienden, buren, collega's, zich vaccineren tegen corona?

14b. Is dat wat uw omgeving kiest van invloed op uw keuze om u te laten vaccineren?

14c. Zo ja, welke mensen zijn voor jou daarin belangrijk?

15a. Heeft u nog overwogen om u wel te laten vaccineren?

15b. Zo ja, wat waren dan redenen om het wel te doen?

16. Welke reden(en) gaf de doorslag om u niet te laten vaccineren?

17a. Heeft u gebruik gemaakt van informatiebronnen bij uw keuze om u niet te laten vaccineren?

17b. Zo ja, welke?

18. Is er een situatie denkbaar waarbij u zich wel zou laten vaccineren? (alternatieve formulering: Zou er iets zijn waardoor u zich wel zou laten vaccineren?)

Respondent heeft er <u>nog niet over nagedacht</u> of hij/zij zich wil laten vaccineren

19. Kunt u toelichten waarom u er nog niet over nagedacht heeft?

20. Wat zou u helpen om straks tot een keuze te komen?

21a. Heeft u behoefte aan bepaalde informatie om tot een keuze te komen?

21b. Zo ja, waarover? *(voor de interviewer: vraag goed door. Wil de respondent bijv. meer weten over de effectiviteit van het vaccin, of de veiligheid van het vaccin, of de instantie/professional die het vaccin straks gaat toedienen, of andere praktische issues? Vraag door!)*

22a. Via welke (media) kanalen zou u deze informatie graag willen ontvangen?
(voor de interviewer: bij kanalen gaat het bijv. om krant, website overheid, social media, talkshow).

22b. Via welke bronnen zou u deze informatie graag ontvangen?
(voor interviewer: wees zo precies mogelijk, vraag door bij antwoord 'overheid' (gaat het om VWS, GGD, RIVM, gemeente etc.? Andere vb antwoorden: eigen huisarts, thuiszorg professional, dominee/imam).

Respondent weet nog niet of hij/zij zich laat vaccineren > twijfelaars

Blokje vragen over redenen voor twijfel

23. Kunt u toelichten waar de twijfel dan vooral zit?
> *(Voor de interviewer: probeer er achter te komen of de twijfel te maken heeft met een pragmatisch argument, bijv. is vaccineren wel nodig als een groot deel al gevaccineerd is straks) of meer een fundamentele oorzaak heeft, dus met conflicterende waarden/principes te maken heeft, zoals solidariteit (reden om het wel te doen) versus persoonlijke integriteit/vrijheid (ik ben niet bang om ziek te worden en wil daarom niet iets in m'n lijf laten spuiten).*

24a. Wat zijn voor u redenen om het wel te doen?
(voor de interviewer: vb antwoorden: zichzelf beschermen, anderen beschermen, corona-maatregelen kunnen eindigen, geanticipeerde spijt)

24b. En wat zijn redenen om het niet te doen?
(voor de interviewer: vb antwoorden: angst voor bijwerkingen, twijfels over effectiviteit, zorgen over veiligheid door snelheid ontwikkeling).

25a. Laten de mensen in uw omgeving, denk aan familie, vrienden, buren, collega's, zich vaccineren tegen corona?

25b. Is dat wat uw omgeving kiest van invloed op uw keuze om u te laten vaccineren?

25c. Zo ja, welke mensen zijn voor jou daarin belangrijk?

26a. Speelt de hoogte van de vaccinatiegraad in Nederland op het moment dat u wordt uitgenodigd nog een rol voor uw besluit om zich te laten vaccineren?

26b. Zo ja, is een hoge vaccinatiegraad dan juist een motivatie om het wel te doen of juist niet?

Blokje vragen over informatiebehoeften

27a. Heeft u al gezocht naar informatie over vaccineren?

27b. Naar welke informatie zocht u?

27c Waar heeft u informatie gezocht? *(voor de interviewer: zocht de respondent op de RIVM website, of website huisarts, of social media, of werd een bekende geraadpleegd etc.)*

27d. Heeft u zoektocht u iets opgeleverd? *(voor de interviewer: ga na of respondent de gezochte informatie kon vinden en als dat zo was of de informatie duidelijk/eenduidig was etc. Vraag door!)*

28a. Zit u nog met bepaalde vragen waar u graag een antwoord op wilt alvorens een keuze te kunnen maken?

28b. Zo ja, wat voor vragen zijn dat?

28c. Bij welke persoon of organisatie zou u graag met uw vragen terecht willen?

28d. Zijn er nog (andere) bronnen waar u graag informatie van wilt ontvangen? *(voor interviewer: wees zo precies mogelijk, vraag door bij antwoord 'overheid' (gaat het om kabinet, VWS, GGD, RIVM, gemeente etc.? Andere antwoorden: eigen huisarts, thuiszorg professional, dominee/imam).*

28e. En heeft u nog een voorkeur via welke kanalen u graag informatie wilt ontvangen? *(voor de interviewer: hangt samen met vorige vraag over bron, maar hier gaat het specifek over het kanaal: brief die thuis bezorgd wordt, website RIVM, social media).*

Toekomstperspectief (vragen voor alle respondenten)

INTRODUCTIETEKSTJE: *6 januari zijn we gestart met vaccineren in Nederland. De komende maanden zullen steeds meer mensen gevaccineerd worden.*

29. Denkt u dat het nodig is dat we de komende maanden de basisregels (zoals 1,5m afstand houden, geen handen schudden, handen wassen) blijven hanteren, terwijl steeds meer mensen gevaccineerd worden?

30. Wat vindt u er van als de komende maanden nog steeds de basisregels (1,5m afstand, geen handen schudden, handen wassen) gelden, terwijl steeds meer mensen gevaccineerd worden?

INTRODUCTIETEKSTJE: *Sommige experts verwachten dat we nog wel een tijd maatregelen nodig hebben tegen het coronavirus, ook als steeds meer mensen straks gevaccineerd zijn.*

31a. Welke maatregelen denkt u nog een paar maanden vol te houden?

31b. Kunt u dat toelichten?

31c. Welke maatregelen denkt u niet voor langere tijd meer vol te houden?

31d. Kunt u dat toelichten?

32. Wat zou u ervan vinden als gevaccineerden straks meer vrijheden krijgen dan niet-gevaccineerden, bijvoorbeeld dat gevaccineerden wel het theater of een sportwedstrijd mogen bezoeken en niet-gevaccineerden niet?

INTRODUCTIETEKSTJE: *De zorgmedewerkers en kwetsbare groepen zijn straks als eerste gevaccineerd.*
33a) Denkt u dat het zinvol is als andere mensen die niet tot de zorgmedewerkers of kwetsbaren behoren zich daarna ook laten vaccineren?
33b) Kunt u dit toelichten?

Afsluitende vraag

We zijn aan het einde gekomen van het interview.

Zijn er nog onderwerpen of vragen waarover u graag iets wilt zeggen?

Vond u het een zinvol gesprek? Heeft dit gesprek u nog aan het denken gezet?

Bedankt voor uw deelname aan dit interview!

[STOP DE BAND]

Verwijs voor inhoudelijke vragen naar de volgende website van de Rijksoverheid:
https://www.rijksoverheid.nl/onderwerpen/coronavirus-vaccinatie

S3. Thematic analysis of interviews about COVID-19 vaccination beliefs

Purpose

We set out to determine the attitudes and opinions which play a role when people are deciding whether or not to get vaccinated against the coronavirus. We did this by examining people's questions and doubts in relation to the pro-vaccination informational campaign.

Methods

Telephone interviews (11 to 14 January 2021) with 60 respondents completed by 8 interviewers, following topic guide (S3). Interviews were transcribed and a thematic analysis was performed.

Results

Continuum: a wide range of perspectives

In the open-ended answers taken from Measurement 8 in mid-November (Appendix 1) and in the interviews, a wide range of responses and feelings were expressed about the vaccine. These varied from relief to suspicion. One striking aspect was that the vaccination willingness of the interviewees could not be divided into clear categories, but had to be placed along a spectrum so that varying attitudes and opinions could be expressed throughout the entire range. At one end, negative arguments dominated and respondents indicated that they 'definitely do not' want to be vaccinated. As the share of positive arguments increases along the spectrum, respondents tend to move from 'have some doubts and probably not', to 'have some doubts but probably yes, and finally to 'definitely yes'. It is important to note that even people who said that they were very willing to be vaccinated, clearly still had concerns about various matters, including the uncertainty surrounding side-effects. A change in some attitudes (e.g., wait-and- see approach) or information could lead to a change in vaccination willingness. The relevant attitudes that were identified in the interviews are explained below.

Arguments in favour of vaccination

The most common reasons expressed by interviewees in favour of vaccination were to protect others. This was usually the most important reason put forward by people who wanted to be vaccinated. These were also the most common reasons given in the open-ended questions asked in the Measurement 8 questionnaire (see Appendix 1). The next most commonly-mentioned reason put forward by the interviewees was that they are doing it for the 'greater good', in other words 'contributing to a way out of the crisis'. Furthermore, the interviewees mentioned as reason to get vaccinated that they could then engage in more social contacts and hug other people, or that they could take part in more activities again during their free time.

Comparison with other vaccines

Some of the interviewees stated that they had thought about getting vaccinated or not, and had come to the conclusion that it was no different than it is with other vaccines where you 'simply get vaccinated' for a trip to a foreign country or for the annual flu jab. This was also frequently mentioned by respondents in the Round 8 questionnaire (mid-November) as a reason for their willingness to be vaccinated against COVID-19.

"It's actually rather strange that, before going on holidays to a foreign country, you simply go to the municipal health service for a vaccination without really knowing exactly what you are letting them put into your body, whereas it has now become such a big issue in a much more urgent situation."

Trust in institutions

Finally, a small number of respondents in the interviews and questionnaires stated that they had confidence that the government was acting for the good of society and that the scientific community was acting responsibly and carefully.

Arguments against vaccination

The most common reason put forward for not (yet) being willing to be vaccinated was uncertainty about the long-term consequences of the vaccines. This is reflected, for example, in respondents' doubts about the effectiveness of the vaccine and the duration of its effectiveness. For example, some respondents were uncertain about whether the vaccine is also effective against the mutated variants of the virus, such as the British and South African variants, and how long the vaccine provides protection.

"If it is only effective for three months, then I won't get it."

In addition, there were also concerns about potential (as yet unknown) long-term side-effects. In this context, some people referred to examples of other medications which were discovered to have negative side-effects over the long term. Young women expressed concerns about the potential effect on fertility and, therefore, their future chances of having children; this was a strong argument against being vaccinated.

"If I personally have to weigh up the potential consequences for my fertility against the fact that I am working with a vulnerable target group, then my wish to have children would be the deciding factor. But I find it a very difficult decision, and it also feels selfish."

Some interviewees also expressed concerns about the direct personal negative consequences of the vaccination in relation to the use of medication and/or having a medical condition. Such concerns primarily had to do with a lack of knowledge about the side-effects of the vaccine in relation to their specific medical condition. For a number of interviewees who had a medical condition, a recommendation from the specialist was the determining factor.

"I let myself be guided by the recommendation of my cardiologist. I am not against vaccinations, but I am also a heart patient and any potential side- effects are, therefore, of critical importance."

Sources of concern

Concerns about the long-term effects seem partly related to the rapid development of the vaccine, the speed of which has raised questions about the safety of the vaccine and any potential (long-term) side-effects. However, concerns regarding the safety of the vaccine have diminished since the end of October.

A few interviewees also indicated that they were specifically distrustful of the mRNA vaccines, as these, in their opinion, were very new and had not yet been sufficiently tested. Others expressed doubts because they would not know beforehand which brand of vaccine

they would be getting. In their opinion, attention should be specifically focused on which vaccine is the least risky for which people. Some interviews made it clear that there is a need for choice in terms of the brand of vaccine and for a form of coordination, for example with the general practitioner, regarding the vaccine.

Various interview and questionnaire respondents indicated that risk perception played a role, and that they expected that they would be fairly resistant to the

coronavirus anyway. This was a reason why some people did not feel that they needed to be vaccinated themselves, and some indicated that they considered the risks associated with the vaccination to be more serious than the risks associated with being infected by the virus.

Distrust in institutions

Finally, distrust of the government, or the pharmaceutical industry, was a reason for a decreased vaccination willingness among some respondents.

Wait-and-see approach

Some of the interviewees had a wait-and-see approach with regard to the vaccination campaign. They indicated that they did not mind standing further back in the vaccination queue. Main reason is the expectation that more knowledge would become available regarding (long-term) side-effects, effectiveness in general, duration of effectiveness, and vaccine safety.

"I find it a challenging step and am happy that it will not be my turn for some time yet. I prefer to wait and see for a while until more is known about the vaccine and what its consequences will actually be. When that becomes clear, I will join the queue but somewhere in the rear."

This attitude was also evident in the open-ended answers: a significant proportion of the respondents weighed up the effectiveness of the vaccine against the potential long-term side-effects (risk assessment).

Decision to be vaccinated or not is not yet necessary

In addition, various interviewees indicated that the situation was not urgent enough for them to make a decision now, or that they had not yet thought 'all that much' about being vaccinated, as the vaccine would not be available for them for some time still. They would decide whether or not to be vaccinated only when the invitation arrived in the post. These people indicated that they needed information that was not yet available, such as the long-term effects and (possibly differing) effects of different vaccines. However, some said that they expected increased knowledge about the vaccine over time to result in greater confidence and, therefore, also an increased willingness to be vaccinated.

"I am not really thinking much about it yet, as it is not my turn yet. It is not relevant for me at the moment. And if I do look for information about it now, then what I read - especially on Facebook - is that there are lots of things that are still uncertain. If it's my turn and I still have doubts, then I'll look it up then. I'm not going to stress out about it now."

Vaccination coverage cuts both ways

For respondents, vaccination coverage in the future seems to be a reason for being vaccinated as well as a reason for not being vaccinated. A higher degree of vaccination coverage reduces the concerns about the long-term effects.

“The more people that are vaccinated, the safer it is for me to also be vaccinated.”

But vaccination coverage also influences the need people felt to also get vaccinated. Within that context, a higher level of vaccination coverage could be a reason for not doing it, and a lower level of vaccination coverage could be a reason for actually getting vaccinated.

“If I see that few people are willing to get vaccinated, then I would be more likely to get it myself, because I could make a bigger difference then.”

Vaccination coverage, therefore, seems to influence people’s perceptions regarding safety on the one hand as well as the social significance of one’s own vaccination on the other.

Information needs

Questions and uncertainties

Interviewees had searched for information about the above-mentioned concerns such as the effect of mRNA vaccines, the effect on fertility, long-term consequences, vaccine safety in relation to vulnerable health, duration of effectiveness and vaccine coverage.

Information sources

Although not everyone indicated that they were already (actively) searching for information, those who had did so by looking at the TV (news programmes such as Op1 and Jinek or the eight o’clock news), Facebook, newspapers, and asking acquaintances (for example, medical professionals or teachers). The special programme on the coronavirus broadcast on 5 January was mentioned by most of the viewers in a positive light: *”it removed my doubts”,* but at the same time it reinforced some people’s negative opinion as they described it as *”the big good news vaccination show”*.

Cue to action

For many of the interviewees who indicated that they had switched from previously being in doubt, to being positive and saying yes to the vaccine, there was a clear *cue to action* – this was provided particularly by TV programmes with doctors which convinced them. These kinds of informative programmes (and also newspaper articles) provided people with more knowledge and confidence about the vaccine, which helped them to make a choice.

”If I see a reputable doctor on TV who answers a number of questions, then it makes me more relaxed and, despite my doubts, also gives me confidence to be vaccinated.”

Medical condition

Interviewees with a medical condition waited for information from their treating physician, or from the interest group that provided information to the specific patient group in question (for example, the Anticoagulation Clinic or Heart Foundation).

Information provision

Respondents with specific questions would like to be able to contact a number of actors such as the general practitioner, treating physician/medical specialist, municipal public health service, or find reliable information on websites of the RIVM (National Institute for Public Health and the Environment), the EMA (European Medicines Agency) or the relevant Dutch counterpart. In addition, various suggestions were made for providing information such as including a

(medical) information leaflet with the invitation, letters for specific risk groups, and visual information for people with limited reading ability. Providing information with the invitation letter was the most popular suggestion by far.

S4. Demographic characteristics of participants in Wave 10 and 11 of the national cohort survey

	Wave 10 (n=54,363)	Wave 11 (n=47,254)	Included in regression analysis (n=3,383)
Gender			
Male	19,826 (36.5%)	17,694 (37.4%)	946 (28.0%)
Female	34,483 (63.4%)	29,533 (62.5%)	2,437 (72.0%)
Other	54 (0.1%)	27 (0.1%)	0
Age			
70+	10,135 (18.6%)	9,550 (20.2%)	358 (10.9%)
55-69	18,706 (34.4%)	16,710 (35.4%)	996 (29.4%)
40-54	15,105 (27.8%)	12,786 (27.1%)	1,201 (35.5%)
25-39	8,931 (16.4%)	7,264 (15.4%)	730 (21.6%)
16-24	1,486 (2.7%)	944 (2.0%)	88 (2.6%)
Education			
Higher education	32,309 (59.4%)	28,781 (60.9%)	1,868 (55.2%)
General upper secondary education	15,043 (27.7%)	12,600 (26.7%)	1,077 (31.8%)
Primary education and general lower secondary education	6,368 (11.7%)	5,326 (11.3%)	438 (12.9%)
Unknown/missing	643 (1.2%)	547 (1.2%)	0
